# Comparison of WHO and CDC growth charts for defining weight status in the young population in Israel: a population-based cross-sectional study

**DOI:** 10.1186/s13584-025-00699-z

**Published:** 2025-06-16

**Authors:** Michal Yackobovitch-Gavan, Moshe Phillip, Shlomit Shalitin

**Affiliations:** 1 The Jesse Z. and Sara Lea Shafer Institute of Endocrinology and Diabetes, National Center for Childhood Diabetes Schneider Children’s Medical Center of Israel, 14 Kaplan St., 49202-35 Petah Tikva, Israel; 2 Department of Epidemiology and Preventive Medicine, School of Public Health, Gray Faculty of Medicine and Health Sciences, Tel Aviv University, Tel Aviv, Israel; 3 Gray Faculty of Medicine and Health Sciences, Tel Aviv University, Tel Aviv, Israel

**Keywords:** BMI z-scores, CDC growth chart, WHO growth charts, Pediatric patients, Weight-status classification

## Abstract

**Background:**

In Israel, the absence of national growth charts leads to the use of CDC or WHO growth charts to assess pediatric growth indices. This population-based cross-sectional study compared BMI z-scores and weight classifications using CDC and WHO charts in children insured by Clalit Health Services (CHS).

**Methods:**

The study analyzed the CHS electronic database for patients aged 2–18 years with at least one recorded BMI measurement from January 2017 to December 2023. Exclusion criteria included improbable BMI measurements (> 60 kg/m^2^ or < 10 kg/m^2^). Demographic data, height, and weight were collected, and BMI z-scores were calculated using both CDC and WHO growth charts. Results were stratified by sex and age groups (2–5 years and > 5–18 years).

**Results:**

The cohort included 1,475,543 children: 488,008 aged 2–5 years (52% male) and 987,535 aged > 5–18 years (51% male). In the younger group, the median CDC BMI z-scores were below 0 (< 50th percentile), while the median WHO BMI z-scores were above 0 for both sexes, with significant differences between methods (*P* < 0.001). BMI z-scores were lower in males than in females across both methods (*P* < 0.001). In the older group, for both methods, the median BMI z-scores were above 0. WHO z-scores had higher medians in males compared to CDC z-scores (*P* < 0.001), while in females, z-scores were similar between methods (*P* = 0.210). CDC showed lower median z-scores for males compared to females (*P* < 0.001), whereas WHO results were comparable between sexes (*P* = 0.337). There were significant discrepancies in weight classification, particularly in the 2–5 age group. Overweight rates were over 4% higher using CDC charts compared to WHO (*P* < 0.001), with minimal agreement (Kappa = 0.06 for males, 0.01 for females). In the older group, WHO classified 4% more children as overweight than CDC (*P* < 0.001), with moderate agreement in males (Kappa = 0.74) and strong agreement in females (Kappa = 0.81).

**Conclusions:**

The study underscores the risk of misclassifying childhood overweight and obesity depending on the growth standard used, particularly in younger children. Policymakers should carefully choose appropriate standards and consider developing national growth charts tailored to the local pediatric population, while allocate resources for early interventions addressing both undernutrition and overnutrition.

**Supplementary Information:**

The online version contains supplementary material available at 10.1186/s13584-025-00699-z.

## Background

Growth charts were created based on longitudinal and/or cross-sectional studies with samples of children and adolescents considered a reference or standard. The growth charts express distributions in percentiles or Z scores and are considered quite sensitive for the assessment of nutritional status, enabling interventions and prevention of health problems. Different growth charts have been proposed by some organizations over the years for use in the world population, through studies with national or international samples and with different inclusion criteria. Among these, are the growth charts constructed by the World Health Organization (WHO) (2006/2007) [1] and the Centers for Disease Control and Prevention (CDC) (2000) [2].

The WHO growth charts for children under the age of five were developed in 2006 based on the Multicenter Growth Reference Study, whose goal was to describe the growth of healthy children [1]. This work was conducted in six countries: Brazil, United States, Ghana, Norway, India and Oman with children considered standard, who lived in socio-environmental and economic conditions ideal for an adequate development [1]. These growth charts were constructed based on longitudinal (from birth to two years old) and cross-sectional samples with children aged two to five years [1]. For children aged five years or more and adolescents aged up to 19 years, the construction of the growth charts was based on the cross-sectional study of the National Center for Health Statistics (NCHS/1977), whose only study population was from the United States [3].

The CDC growth charts were drawn up in the 2000 s based on five national surveys conducted in the United States [2, 4]. They are expressed in percentiles and are specific by sex and age group.

Body mass index (BMI), is a useful index for measurement of optimal physical growth in children. BMI norms are established in the WHO and CDC growth charts [1, 2]. However, WHO and CDC have different base population, leading to different growth-charts and cut-offs. In fact, previous studies have shown that for a given population, WHO-BMI-z scores are associated with increased percentage of overweight and obesity and reduced percentage of underweight when compared to CDC-BMI-z scores [5–9].

Israel health authorities did not develop national growth charts. Researchers and clinicians in Israel evaluate height, weight, and BMI status based on the U.S. CDC or WHO growth charts.

Previous publications have assessed and validated the applicability of WHO and CDC growth charts to evaluate growth in Israeli children [10]. Others have assessed the appropriateness of U.S. and international BMI-for-age references to define adiposity among Israeli school children [11]. Recently, Gabbay et al. [12] reported that BMI in Israeli children was significantly higher compared to the CDC and WHO consistently along the age range.

The primary aims of this population-based cross-sectional study were: 1. to compare the distribution of BMI z-scores using the CDC vs. WHO growth chart data in pediatric patients (aged 2–18 years) insured by Clalit Health Services (CHS), and 2. to compare the rates of pediatric patients classified as underweight, normal weight, overweight, and obese based on the BMI z-scores using each of these references.

The secondary aims were to compare BMI z-scores and weight status categories based on these references between males and females, and to identify which of these growth charts is more suitable for use in the young population of Israel.

## Methods

This cross-sectional study was conducted using the electronic database of Clalit Health Services (CHS), an Israeli payer-provider integrated health care system. CHS is the largest health maintenance organization in Israel, serving ~ 54% of the national population. The database is accumulated by continuous real-time input from physicians and health service providers, and includes patient demographic, socioeconomic, and clinical characteristics, hospital discharge and outpatient clinic diagnoses, laboratory test results, medical treatments, and medication dispensation information.

Data was extracted from Clalit Health Services (CHS) using Clalit’s Data sharing platform powered by MDClone (https://www.mdclone.com).

### Study population

CHS is using WHO growth charts for children aged 0–19 years. The electronic database of CHS was searched for all the patients aged 2–18 years with at least one documented BMI value at a visit with a PCP between January 2017 and December 2023. Study exclusion criteria were improbable measurements, BMI > 60 kg/m^2^ or BMI < 10 kg/m^2^.

### Data measurements and variables

From the medical files, demographic parameters (sex, age, origin, socioeconomic position) were retrieved. Data collected during the study period included anthropometric parameters (height and weight as measured by nurses or PCP, and calculated BMI).

BMI was calculated as weight (in kilograms) divided by height (in meters) squared. To compare BMI values across age groups by sex, BMI-Z scores were calculated using the WHO [13, 14] and the CDC [15] growth charts.

The WHO definitions for weight status for children are based on sex- and age-specific BMI Z scores [13, 14]. For children aged 0 to 5 years child BMI categories definitions are: underweight: BMI- Z score < − 2, normal weight: − 2 ≤ BMI- z score < 2, overweight: 2 ≤ BMI-Z score ≤ 3 and obesity: BMI- Z score > 3. For children aged 5 to 19 years, the BMI categories definitions are: underweight: BMI- z score < −2, normal weight: −2 ≤ BMI- z score < 1, overweight: 1 ≤ BMI- Z score ≤ 2, and obesity: BMI- Z score > 2.

The CDC definitions for weight status for children are based on sex- and age-specific percentiles [15]. According the CDC, child BMI categories definitions are: underweight: BMI < 5th percentile (BMI-Z score < −1.645), normal weight: 5th percentile < BMI > 85th percentile percentiles (− 1.645 ≤ BMI-Z score ≤ 1.036), overweight: 85th percentile < BMI > 95th percentile (1.036 < BMI-Z score ≤ 1.645), and obesity: BMI > 95th percentile or greater (BMI-Z score > 1.645).

The study was approved by the local institutional ethics committee in keeping with the principles of the Declaration of Helsinki. In accordance with the Ministry of Health regulations, the institutional ethics committee did not require written informed consent, as data were collected anonymously from computerized medical files, with no active participation of patients.

### Statistical analysis

Statistical analyses were performed using SPSS software, version 29 (SPSS, Inc., Chicago, Illinois).

Data are presented as n (%) or median (interquartile range, IQR, for skewed distributions). All analyses are stratified according to age group (2–5 years and > 5–18 years).

BMI-Z score according to the WHO and the CDC-2000 growth curve data within each age and sex group, were compared using paired samples Wilcoxon signed-rank test (skewed distribution).

Weight status categories (underweight, normal weight, overweight and obesity) were recoded according to the WHO and the CDC-2000 definitions [13–15], and the ratios of each of the weight status categories were compared between the WHO and the CDC-2000 using Chi-square tests. Kappa coefficients were calculated for each of the weight status categories to evaluate the extent of agreement between frequencies according to the WHO and the CDC-2000 definitions.

Between sexes comparisons were conducted using Mann Whitney U-test for numerical variables with skewed distribution, or Chi-square tests for categorial variables.

## Results

**Fig. 1:**
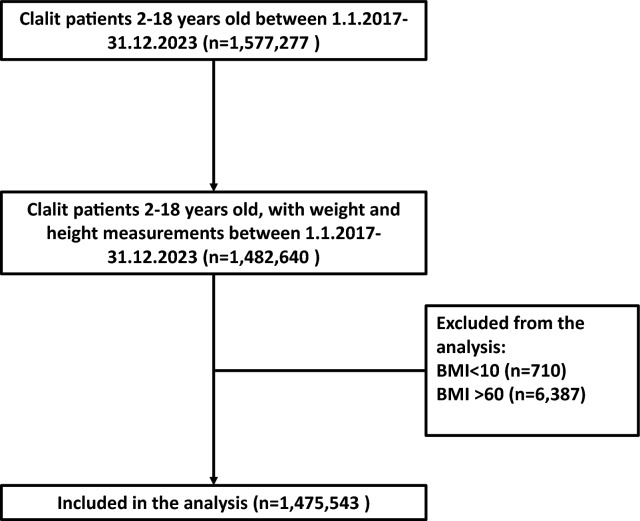
Flow-chart of the study participants

During the study period, 1,577,277 children (51.5% male), aged 2–18 years (median 6.9 years; interquartile range 4.3 to 13.1 years), were insured by CHS. Of these, 1,482,640 (94%) had at least one recorded BMI measurement. After excluding those with improbable measurements (6,387 with a documented BMI > 60 kg/m^2^ and 710 with BMI < 10 kg/m^2^), the final cohort included 1,475,543 children (Fig. 1).

The cohort comprised 71.6% Jews and 28.4% Arabs. Socioeconomic position (SEP), according to the Israel Central Bureau of Statistics [16], was distributed as follows: 27.5% in the low SEP group, 54.7% in the medium SEP group, and 17.7% in the high SEP group. The majority of Arab children in the study belonging to the low SEP group (74.1% of Arab participants, compared to 11.0% of Jewish participants), while the majority of Jewish children belonging to the medium SEP group (65.1% of Jewish participants, compared to 25.2% of Arab participants), *P* < 0.001. Additionally, Arab children in our study had higher BMI z-scores compared to Jewish children [median BMI z-score according to the CDC: 0.15 (IQR: − 0.77, 0.94) for Arab children vs. 0.04 (IQR: − 0.80, 0.84) for Jewish children; according to the WHO: 0.24 (IQR: − 0.63, 1.09) for Arab children vs. 0.18 (IQR: − 0.61, 1.02) for Jewish children; *P* < 0.001] (Supplementary Table 1).

In our cohort, 488,008 participants were aged 2–5 years, of whom 255,407 (52%) were male; and 987,535 participants were aged > 5–18 years, of whom 505,071 (51%) were male.

Table 1 presents comparison of BMI-Z score according to the CDC 2000 and WHO growth charts between males and females, stratified according to age groups.

**Table 1:** BMI-Z according to the CDC and WHO growth-charts: Comparison between sexes, stratified by age group

	Males	Females	P
*Age 2–5 years*			
n	255,407	232,601	
Age, years			
Median (IQR)	2.7 (2.2, 3.7)	2.7 (2.2, 3.7)	0.678
BMI-Z CDC			
Median (IQR)	− 0.24 (− 1.12, 0.55)	− 0.11 (− 0.97, 0.66)	< 0.001
BMI-Z WHO			
Median (IQR)	0.10 (− 0.67, 0.86)	0.16 (− 0.59, 0.89)	< 0.001
*Age* > *5–18 years*			
n	505,071	482,464	
Age, years			
Median (IQR)	11.5 (6.7, 14.0)	11.5 (6.7, 14.1)	0.555
BMI-Z CDC			
Median (IQR)	0.16 (− 0.73, 0.99)	0.24 (− 0.56, 0.99)	< 0.001
BMI-Z WHO			
Median (IQR)	0.24 (− 0.65, 1.19)	0.24 (− 0.57, 1.10)	0.337

Data are presented as median (interquartile range, IQR) (skewed distribution)

P value represents the difference between males and females using Mann–Whitney U-test

Median age in the younger group was 2.7 years (IQR 2.2, 3.7) and in the older group 11.5 years (6.7, 14.0).

Compared to the reference population’s BMI z-score median and interquartile range (IQR) of 0 (− 0.675, 0.675), which reflects a median at the 50th percentile and an IQR at the 25th and 75th percentiles, the younger group (2–5 years) had a median BMI z-score below 0 according to the CDC and a median BMI z-score above 0 according to the WHO for both sexes. There were significant differences between the medians of the two methods (*P* < 0.001 for both sexes). In both methods, BMI Z-scores were lower in males compared to females (*P* < 0.001).

In the older group (> 5–18 years), we observed a median BMI z-score above 0 according to both methods. Among males, the WHO BMI Z-scores showed a higher median and IQR compared to the CDC BMI Z-scores (*P* < 0.001). In females, BMI Z-scores were comparable between the two methods (*P* = 0.210). According to the CDC-2000, males had a lower median BMI Z-score compared to females (*P* < 0.001), while according to the WHO, BMI Z-scores were comparable between the sexes (*P* = 0.337).

**Fig. 2:**
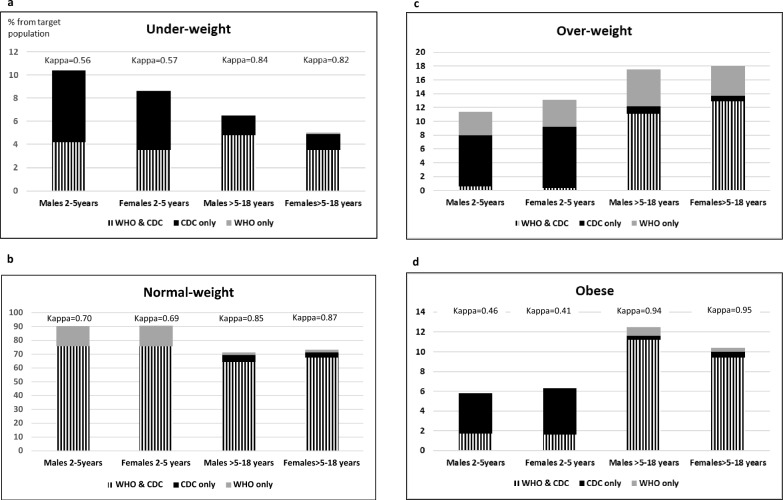
Comparison of weight-status category rates according to WHO and CDC, stratified by age-group and sex. **a** Underweight, **b** normal-weight, **c** over-weight, **d **Obese

Table 2 and Fig. 2 present comparisons of the rates of weight status categories according to the WHO and CDC definitions, stratified by age group and sex. The comparisons of the rates of weight status categories between the two methods show significant discrepancies, especially in the younger age group.

**Table 2:** Rates of weight-status categories according to the WHO and CDC, stratified by age-group and sex

	CDC	WHO	P1
*Age 2–5 years (n* = *488,008)*			
Under-weight, n (%)			
Males	26,341 (10.3)	10,611 (4.2)	< 0.001
Females	19,947 (8.6)	8132 (3.5)	
P2	< 0.001	< 0.001	< 0.001
Healthy-weight, n (%)			
Males	193,833 (75.9)	230,182 (90.1)	< 0.001
Females	176,345 (75.8)	210,577 (90.5)	
P2	0.068	0.157	< 0.001
Over-weight, n (%)			
Males	20,480 (8.0)	10,189 (4.0)	< 0.001
Females	21,539 (9.3)	10,097 (4.3)	
P2	< 0.001	0.219	< 0.001
Obesity, n (%)			
Males	14,753 (5.8)	4425 (1.7)	< 0.001
Females	14,770 (6.3)	3795 (1.6)	
P2	< 0.001	0.009	< 0.001
*Age* > *5–18 years (n* = *987,535)*			
Under-weight, n (%)			
Males	33,190 (6.6)	24,408 (4.8)	< 0.001
Females	23,708 (4.9)	17,612 (3.7)	
P2	< 0.001	< 0.001	< 0.001
Healthy-weight, n (%)			
Males	350,810 (69.5)	336,130 (66.6)	< 0.001
Females	344,594 (71.4)	334,281 (69.3)	
P2	< 0.001	< 0.001	< 0.001
Over-weight, n (%)			
Males	62,038 (12.3)	83,164 (16.5)	< 0.001
Females	66,138 (13.7)	83,206 (17.2)	
P2	< 0.001	< 0.001	< 0.001
Obesity, n (%)			
Males	59,033 (11.7)	61,369 (12.2)	< 0.001
Females	48,024 (10.0)	47,365 (9.8)	< 0.001
P2	< 0.001	< 0.001	

Data are presented as n (%)

P1 represents *P* value for the difference of the rates of weight status categories between the CDC and WHO growth data using Pearson chi-square test

P2 represents *P* value for the differences between males and females within each weight status category using Pearson chi-square test

In the younger age group, the rates of underweight are more than 5% greater according to the CDC compared to the WHO definitions for both males and females (*P* < 0.001), with a weak agreement between the two methods in classifying underweight (Kappa = 0.56 and 0.57 for males and females, respectively) (Fig. 2a). The rates of normal weight are over 14% greater according to the WHO compared to the CDC definitions for both males and females (*P* < 0.001), with a moderate agreement between the methods in classifying normal weight (Kappa = 0.70 and 0.69 for males and females, respectively) (Fig. 2b). The rates of overweight are more than 4% greater according to the CDC compared to the WHO definitions for both males and females (*P* < 0.001). The rates of overweight showed the largest discrepancy between the two methods, with virtually no agreement (Kappa = 0.06 and 0.01 for males and females, respectively), and only 0.4% of males and 0.6% of females in the younger age group were classified as overweight by both methods (Fig. 2c). All children classified as overweight only by the CDC (7.4% of males and 8.8% of females in the younger group) were classified as having normal weight by the WHO definition. Conversely, all children classified as overweight only by the WHO (3.4% of males and 3.9% of females in the younger group) were classified as having obesity by the CDC definition. The rates of obesity are more than 4% greater according to the CDC compared to the WHO definitions for both males and females (*P* < 0.001), with a weak agreement between the methods in classifying obesity (Kappa = 0.46 and 0.41 for males and females, respectively) (Fig. 2d). Most of the children classified as having obesity only by the CDC were classified as overweight by the WHO definition, and fewer than 1% were classified as having normal weight according to the WHO definition.

In the older age group, the discrepancies are smaller compared to the younger age group; however, significant differences remain in the definitions of overweight. The rates of underweight are more than 1% greater according to the CDC compared to the WHO definitions for both males and females (*P* < 0.001), with a strong agreement between the two methods in classifying underweight (Kappa = 0.84 and 0.82 for males and females, respectively) (Fig. 2a). The rates of normal weight are 1–3% greater according to the CDC compared to the WHO definitions for both males and females (*P* < 0.001), with a strong agreement between the methods in classifying normal weight (Kappa = 0.85 and 0.87 for males and females, respectively) (Fig. 2b). The rates of overweight are about 4% greater according to the WHO compared to the CDC definitions for both males and females (*P* < 0.001). This category showed the largest discrepancy between the two methods, with a moderate agreement in males (Kappa = 0.74) and a strong agreement in females (Kappa = 0.81) (Fig. 2c). Most of the children who were classified as overweight only by the CDC definition (1.1% of males and 0.8% of females in the older group) were classified as having obesity according to the WHO definition. Conversely, most of the children classified as overweight only by the WHO definition (5.3% of males and 4.3% of females in the older group) were classified as having normal weight by the CDC definition. The rates of obesity differ by less than 1% between the CDC and WHO definitions in both sexes, with an almost perfect agreement between the two methods in classifying obesity (Kappa = 0.94 and 0.95 for males and females, respectively) (Fig. 2d).

We also observed differences between males and females regarding the rates of different weight status categories according to both methods. These differences include higher rates of underweight in males compared to females across all age groups (*P* < 0.001), higher rates of overweight in males in the younger group (*P* < 0.001) and in females in the older group (*P* < 0.001), and higher rates of obesity in females in the younger group (*P* < 0.001) and in males in the older group (*P* < 0.001).

## Discussion

The findings of this study provide valuable insights into the discrepancies between the WHO and CDC BMI charts in assessing weight status in children, particularly emphasizing the differences in classification across age and gender.

The analysis of nearly 1.5 million Israeli children aged 2–18 years reveals significant variations, particularly in children under five, where the WHO and CDC standards diverge most notably.

In children (both males and females) aged 2–5 years, the median BMI for Israeli children was below zero according to CDC standards but above zero according to WHO standards. In the older children (both males and females), both methods showed BMI Z-scores above zero. Our results in the older age group (> 5–18 years) are consistent with the results of a recent study based also on anthropometric data from Clalit Health Services that was conducted in a smaller cohort of 15,650 Israeli children aged 4–18 years [12]. That study reported that Israeli children had significantly higher BMI values than both the WHO and CDC reference charts across the entire age range, indicating a concerning trend of increased adiposity in Israeli children [12]. However, that study evaluated only children older than four years and did not analyze children aged 2–5 years separately, who have distinct growth charts and different weight status cutoffs according to the WHO standards [12].

We found mild sex differences in the discrepancies between the two methods. For all boys and for girls under five years, the WHO standards yielded higher median BMI Z-scores compared to the CDC, while the scores for girls five years or older were similar across both methods. Additionally, the median BMI Z-score according to the CDC was lower for males compared to females, whereas the median BMI Z-score according to the WHO was lower for males only in children under five years, and comparable between males and females in the older age group. These results indicate that both methods diverge from the Israeli pediatric population’s BMI Z-score distribution. The findings regarding sex differences align with the results of the previous Israeli study [12], that showed that both Israeli boys and girls had significantly higher BMI values compared to the WHO and CDC reference charts. Moreover, the rate of statistically significant differences in age-matched BMI was slightly higher in girls than in boys [12].

The discrepancies between the methods also extended to weight status categories. The study found that underweight rates were significantly higher across all ages and both sexes using the CDC standards. This may be attributed to the differences in growth references used by each organization, with the CDC tending to classify more children as underweight. This finding is consistent with previous studies that have shown that CDC BMI-Z scores are associated with a higher percentage of underweight classifications compared to WHO BMI-Z scores in different pediatric populations worldwide [5–9, 17].

The most striking difference between the methods occurred in the overweight category, where agreement was almost nonexistent in children under five years. The WHO uses stricter criteria for diagnosis of overweight (+ 2 SD) and obesity (+ 3 SD) in children under five years, resulting in fewer children being classified as overweight or obese compared to the CDC. This indicates that the CDC may be more sensitive or possibly overestimating early childhood overweight and obesity. This inconsistency suggests that the use of different growth standards may lead to substantial differences in early intervention rates for childhood overweight, depending on which standard is applied. However, both methods aligned better after age five, where the WHO criteria for overweight (+ 1 SD) and obesity (+ 2 SD) are similar to the CDC criteria. This alignment in older children suggests that both methods converge on a similar interpretation of overweight and obesity of Israeli population as children mature, despite earlier discrepancies. Gender differences were also noted, with higher rates of overweight and lower rates of obesity in males compared to females in the younger age group, and the opposite pattern, with higher rates of overweight and lower rates of obesity in females compared to males, in the older age group. These gender differences may reflect physiological and growth pattern variations that require further exploration in future research.

A systematic review by Oliveira et al., published in 2022, involved 33 studies conducted between 2007 and 2020 and analyzed growth data from various countries. The review indicated that the WHO BMI-for-age charts generally outperformed the CDC charts in assessing underweight, overweight, and obesity, especially for children under five in countries such as the United States, South Africa, and Brazil. WHO charts, developed using multiethnic data, showed broader international applicability due to their comprehensive design. For children over five, the WHO charts continued to perform well in populations such as Brazil and Canada, whereas the CDC charts underperformed compared to WHO charts, often leading to an overestimation of overweight and obesity, particularly in South Africa and Saudi Arabia. However, in countries like Canada, the CDC charts showed closer alignment with WHO references, indicating some regional accuracy. This systematic review, along with our data from a large pediatric Israeli cohort, emphasizes the complexity of establishing a single global standard for growth and nutritional assessments [18].

The strengths of our study include the large cohort of children from the largest health maintenance organization in Israel, the use of objective measurements of BMI, representation across a large age group, and inclusion of different ethnicities (both Arab and Jewish) and diverse socioeconomic clusters.

However, limitations such as the possibility of documenting improbable BMI measurements due to mistakes in recorded height and weight exist, although we excluded invalid measurements from the analysis.

The findings of this study highlight several important health policy considerations, particularly in the context of childhood obesity and underweight classification, which have significant implications for public health interventions, resource allocation, and the overall approach to pediatric care. Given the discrepancies between the WHO and CDC BMI charts, particularly in younger children, this research underscores the need for tailored health policies to better address the unique growth patterns and nutritional needs of children in Israel.

The study highlights the risk of underestimating or overestimating childhood overweight and obesity depending on the growth standards used, particularly in children under five years old. The WHO’s more stringent overweight and obesity cutoffs could result in lower intervention rates, while the CDC’s higher sensitivity might lead to over-intervention. Policymakers must carefully evaluate which standards to apply to ensure that early detection and intervention strategies are appropriately targeted. Policy makers may also consider implementing national growth charts tailored to the local pediatric population. Simultaneously, resources should be allocated for early intervention programs targeting at-risk children, focusing on both undernutrition and overnutrition.

The results from this study, along with other Israeli research, show that children in Israel, especially those over the age of five, tend to have higher BMI values compared to international references and high rates of over-weight and obesity. This underscores the need for robust public health campaigns promoting balanced nutrition and regular physical activity among school-aged children to curb the trend of increasing adiposity. Such campaigns could be implemented through schools, community centers, and online platforms to reach a wide audience.

It is important to mention that in our study population, we found an association between ethnicity and socioeconomic status, with the majority of Arab children in the study belonging to a lower socioeconomic status. This finding is consistent with the latest report (2023) from the Israeli Central Bureau of Statistics, which states that 82% of the Arab population in Israel falls into a low socioeconomic category [19]. Additionally, Arab children in our study had higher BMI z-scores compared to Jewish children. These findings are consistent with previous reports in both adults and children, including our own recent studies [20, 21]. However, analyzing the research question regarding the comparison between the two growth chart systems based on ethnicity and socioeconomic status was not within the scope of our study, as the CDC and WHO growth charts were designed for broad populations without specific consideration of ethnicity or socioeconomic status.

A limitation of this study is the potential for errors in documented height and weight measurements, which may impact the accuracy of BMI calculations. To address this, health policies should emphasize the importance of accurate data collection and standardization across all health facilities, particularly in pediatric care. Investments in training healthcare professionals and improving data recording systems can mitigate such errors.

## Conclusions

This study highlights significant discrepancies between the WHO and CDC BMI charts in assessing weight status among Israeli children, particularly in those under five years, where the WHO’s stricter criteria result in fewer overweight and obesity classifications compared to the CDC. These differences, especially in early childhood, could impact clinical interventions and public health strategies. As children age, the two standards align more closely, though sex differences in weight classification persist. The findings underscore the complexity of applying a single global standard for growth assessments across diverse populations and emphasize the importance of choosing the most appropriate reference standard for accurate pediatric growth and nutritional evaluations.

Clinicians and policy makers worldwide, and specifically in Israel, need to be aware of these differences to ensure that children are accurately assessed and receive appropriate health interventions tailored to their needs. Policy makers may also consider implementing national growth charts tailored to the local pediatric population.

## Supplementary Information

Supplementary material 1.

## Data Availability

The datasets generated and analyzed during the current study belong to the Clalit Health Services and are not publicly available. The data files used for the present study are publicly unavailable according to the privacy regulations of Clalit Health Services.

## Abbreviations

WHO: World Health Organization
CDC: Centers for Disease Control and Prevention
BMI: Body mass index
CHS: Clalit Health Services
SEP: Socioeconomic position
