# Supplementary material for: Comparison of WHO and CDC growth charts for defining weight status in the young population in Israel: a population-based cross-sectional study
Source: Isr J Health Policy Res. 2025 Jun 16;14:37. doi: 10.1186/s13584-025-00699-z (PMC12168255; doi:10.1186/s13584-025-00699-z)
Supplement: Supplementary file 1 — Supplementary material 1. [file 13584_2025_699_MOESM1_ESM.docx]

**Supplementary Table 1**: SEP and BMI-z score: comparison according to ethnicity.

|  | **Jew**  **n=1056489 (71.6%)** | **Arab**  **n=419054 (28.4%)** | **P** |
| --- | --- | --- | --- |
| **SEP (%)**  **Low**  **Medium**  **High**  **missing** | 11.0%  65.1%  23.9%  5.3% | 74.1%  25.2%  0.7%  14.2% | <0.001 |
| **BMI-Z CDC**  **Median (IQR)** | 0.04 (-0.80, 0.84) | 0.15 (-0.77, 0.94) | <0.001 |
| **BMI-Z WHO**  **Median (IQR)** | 0.18 (-0.61, 1.02) | 0.24 (-0.63, 1.09) | <0.001 |

SEP, Socioeconomic position. Data are presented as percent (categorial variable) or median (interquartile range, IQR) (skewed distribution). P value represents the difference between Jews and Arabs using Pearson chi-square test or Mann-Whitney U-test.
